# 3-Methylcholanthrene Induces Chylous Ascites in TCDD-Inducible Poly-ADP-Ribose Polymerase (*Tiparp*) Knockout Mice

**DOI:** 10.3390/ijms20092312

**Published:** 2019-05-10

**Authors:** Tiffany E. Cho, Debbie Bott, Shaimaa Ahmed, David Hutin, Alvin Gomez, Laura Tamblyn, Angela C. Zhou, Tania H. Watts, Denis M. Grant, Jason Matthews

**Affiliations:** 1 Department of Pharmacology and Toxicology, University of Toronto, Toronto, ON M5S 1A8, Canada; tiffany.cho@mail.utoronto.ca (T.E.C.); debb.bott@gmail.com (D.B.); shaimaa.ahmed1@gmail.com (S.A.); dhutin1@gmail.com (D.H.); alvin_v_gomez@yahoo.com (A.G.); Laura.Tamblyn@uhnresearch.ca (L.T.); denis.grant@utoronto.ca (D.M.G.); 2 Department of Immunology, University of Toronto, Toronto, ON M5S 1A8, Canada; angel.zhou@mail.utoronto.ca (A.C.Z.); tania.watts@utoronto.ca (T.H.W.); 3 Department of Nutrition, Institute of Basic Medical Sciences, University of Oslo, Sognsvannsveien 9, 0372 Oslo, Norway

**Keywords:** TCDD-inducible poly-ADP-ribose polymerase (TIPARP), 3-methylcholanthrene, chylous ascites, wasting syndrome, 2,3,7,8-tetrachlorodibenzo-*p*-dioxin

## Abstract

TCDD-inducible poly-ADP-ribose polymerase (TIPARP) is an aryl hydrocarbon receptor (AHR) target gene that functions as part of a negative feedback loop to repress AHR activity. *Tiparp*^−/−^ mice exhibit increased sensitivity to the toxicological effects of 2,3,7,8-tetrachlorodibenzo-*p*-dioxin (TCDD), including lethal wasting syndrome. However, it is not known whether *Tiparp*^−/−^ mice also exhibit increased sensitivity to other AHR ligands. In this study, we treated male *Tiparp*^−/−^ or wild type (WT) mice with a single injection of 100 mg/kg 3-methylcholanthrene (3MC). Consistent with TIPARP’s role as a repressor of AHR signaling, 3MC-treated *Tiparp*^−/−^ mice exhibited increased hepatic Cyp1a1 and Cyp1b1 levels compared with WT mice. No 3MC-treated *Tiparp*^−/−^ mice survived beyond day 16 and the mice exhibited chylous ascites characterized by an accumulation of fluid in the peritoneal cavity. All WT mice survived the 30-day treatment and showed no signs of fluid accumulation. Treated *Tiparp*^−/−^ mice also exhibited a transient and mild hepatotoxicity with inflammation. 3MC-treated WT, but not *Tiparp*^−/−^ mice, developed mild hepatic steatosis. Lipid deposits accumulated on the surface of the liver and other abdominal organs in the 3MC-*Tiparp*^−/−^ mice. Our study reveals that *Tiparp*^−/−^ mice have increased sensitivity to 3MC-induced liver toxicity, but unlike with TCDD, lethality is due to chylous ascites rather than wasting syndrome.

## 1. Introduction

The aryl hydrocarbon receptor (AHR) is a ligand-dependent transcription factor that mediates a wide range of biological effects in response to endogenous and dietary ligands. Toxicological effects are induced upon activation by numerous environmental and synthetic ligands, including 2,3,7,8-tetrachlorodibenzo-*p*-dioxin (TCDD) and polycyclic aromatic hydrocarbons (PAHs) such as 3-methylcholanthrene (3MC) [1]. In response to 3MC or other AHR agonists, the AHR translocates to the nucleus where it heterodimerizes with AHR nuclear translocator (ARNT) and the complex then binds to AHR response elements (AHREs) located in the 5′ regulatory region of hundreds of genes, including cytochrome P450 1A1 (CYP1A1), CYP1B1, and TCDD-inducible poly-ADP-ribose-polymerase (TIPARP) [1,2].

PAHs are a group of ubiquitous environmental pollutants that are generated from anthropogenic and natural incomplete combustion processes [3]. Important sources include cigarette smoke, diesel exhaust, and grilled foods [4]. The main routes of exposure for humans are via the inhalation of PAHs from the ambient air and through the ingestion of charred foods [5]. The toxic mechanism of action for PAHs includes both their well-established genotoxicity, which results from their bioactivation to mutagenic metabolites that form DNA adducts, and their ability to mediate non-genotoxic effects through various intracellular receptors [6,7]. The metabolism of PAHs is multifaceted with the AHR-mediated induction of drug-metabolizing enzymes, such as CYP1A1 and CYP1B1, being central to the generation of reactive metabolites [3]. 3MC is a synthetic PAH that is genotoxic and mutagenic, and has also been reported to increase cell proliferation, alter reproduction, modulate estrogen signaling, and activate the AHR [8,9,10]. However, unlike TCDD, a single high-dose exposure to 3MC does not cause severe hepatotoxicity or induce wasting syndrome, although mild hepatosteatosis has been reported [11].

TIPARP, also known as poly-ADP-ribose polymerase 7 (PARP7) or ADP-ribosyltransferase diphtheria toxin-like 14 (ARTD14), is an AHR target gene and a member of the PARP family of proteins, which catalyze the transfer of ADP-ribose units onto themselves or onto acceptor proteins [12,13,14]. ADP-ribosylation is a post-translational modification that is involved in numerous cellular processes, including metabolism, DNA repair, immune cell function and regulation, protein stability, and gene regulation [15]. TIPARP is a mono-ADP-ribosyltransferase and it is predominantly localized in the nucleus in many cell types [12,16]. Its cellular localization is dependent on a N-terminal nuclear localization signal and a CCCH-zinc finger domain [17]. TIPARP is involved in several biological processes including innate immunity, responses to viral infection, stem cell pluripotency, astrocyte autophagy, and the regulation of transcription [12,18,19,20]. TIPARP expression is induced by platelet-derived growth factors [21], viral infection [22], nuclear hormone receptors [23] and AHR [2]. Increasing evidence suggests that TIPARP functions as part of a negative feedback loop to regulate AHR signaling through mono-ADP-ribosylation [12,13]. *Tiparp*^−/−^ and hepatocyte-specific *Tiparp*^−/−^ mice treated with a normally non-lethal dose of 10 µg/kg TCDD exhibit increased sensitivity to TCDD-induced toxicities including the development of steatohepatitis, hepatotoxicity, and lethal wasting syndrome [13,24]. However, whether *Tiparp*^−/−^ mice exhibit increased sensitivity to other AHR ligands has not been determined.

In the present study, we treated male *Tiparp*^−/−^ or wild type (WT) mice with a single non-lethal dose of 100 mg/kg 3MC and monitored them for up to 30 days. Interestingly, no 3MC-treated *Tiparp*^−/−^ mice survived beyond day 16. This increased sensitivity to 3MC-induced lethality was not due to severe hepatotoxicity or wasting syndrome, but rather the mice exhibited a chylous ascites condition characterized by the peritoneal accumulation of a viscous fluid with high lipid and protein content. Our data show that TIPARP has an important role in modulating the differential toxic effects of two distinct AHR ligands, and further characterize it as a key regulator of the AHR signaling pathway.

## 2. Results

**Figure 1:**
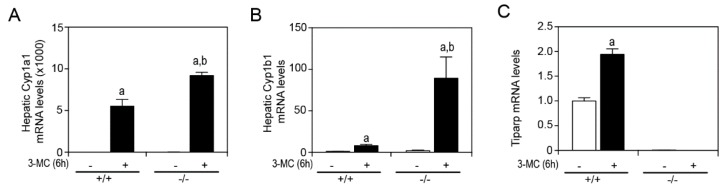
Hepatic gene expression of aryl hydrocarbon receptor (AHR) target genes in male mice 6 h after treatment with 100 mg/kg 3MC. Hepatic RNA was isolated, reverse transcribed and mRNA expression levels of (**A**) Cyp1a1, (**B**) Cyp1b1 and (**C**) Tiparp were determined by qPCR. Data represent the mean ± SEM; *n* = 3 for all genes. ^a^ *p* < 0.05 two-way ANOVA comparison between genotype-matched corn oil- and 3MC-treated mice and ^b^ *p* < 0.05 two-way ANOVA comparison between treatment-matched WT and *Tiparp*^−/−^ mice followed by a Tukey’s post hoc test.

In a previous study by our group, we reported that treatment of male or female *Tiparp*^−/−^ mice with a single intraperitoneal (IP) injection of 100 µg/kg TCDD resulted in an enhanced sensitivity to wasting syndrome causing death between day 3 and 5; treated WT mice survived to the end of the 30-day period [13]. These data provided further support for TIPARP’s role as a negative regulator of AHR activity [12]. To determine if *Tiparp*^−/−^ mice exhibit increased AHR signaling in the presence of another, but readily metabolizable AHR ligand, male *Tiparp*^−/−^ mice and WT mice were treated with a single IP injection of 100 mg/kg 3MC. The hepatic mRNA expression levels of Cyp1a1 and Cyp1b1 were significantly higher in 3MC-treated *Tiparp*^−/−^ mice compared with WT mice after a 6 h exposure (Figure 1A,B). Tiparp mRNA levels were increased in WT but not in *Tiparp*^−/−^ mice (Figure 1C).

**Figure 2:**
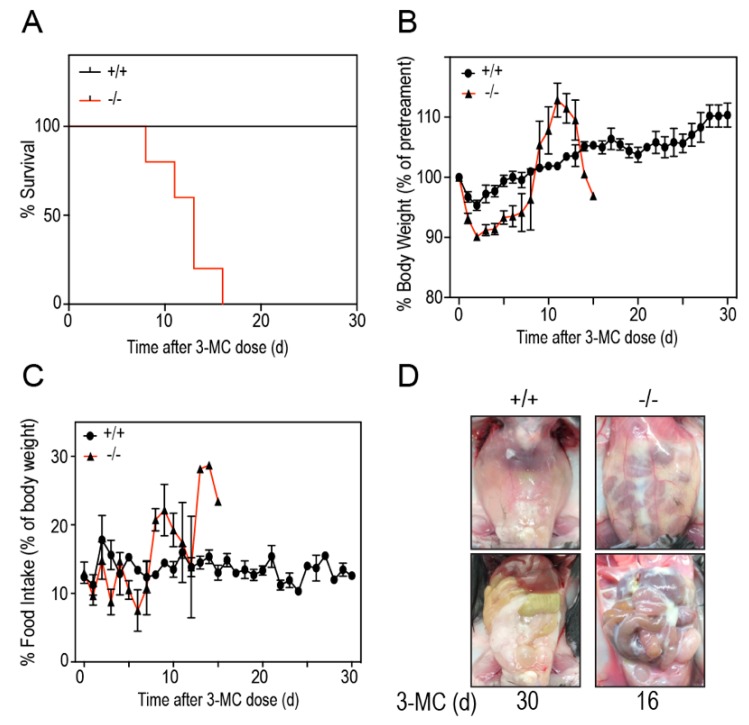
Thirty-day survival study characteristics. (**A**) Kaplan–Meier survival curves indicating the survival rate of 3MC-treated WT (+/+) and *Tiparp*^−/−^ (−/−) mice. Mice were euthanized when body weight loss exceeded 20% of the baseline (day 0) value or if the animal had reached an endpoint as described in the Materials and Methods. (**B**) Daily body weights expressed as a percent of baseline values (i.e., day 0). (**C**) Daily food intake measurements expressed as a gram per gram percentage of daily mouse body weight normalized to baseline values. For B and C, the data represent the mean ± SEM; *n* = 4–6. (**D**) Representative images of the closed peritoneum and opened peritoneal cavity of WT (left; day 30) and *Tiparp*^−/−^ mice (right; day 16). Images on the top show an intact peritoneum with fluid accumulation in the 3MC-treated *Tiparp*^−/−^ mice (right) compared with similarly treated WT mice (left). Images on the bottom show the open abdomen and all tissues in the peritoneal cavity. Reduced epididymal white adipose tissue was observed in the *Tiparp*^−/−^ mice with remnants of the fluid adhering to the tissues.

To study if *Tiparp*^−/−^ mice exhibit increased sensitivity to 3MC-induced toxicity, we treated *Tiparp*^−/−^ and WT mice with a single IP injection of 100 mg/kg 3MC and monitored them for up to 30 days. All WT mice treated with 3MC survived the duration of the study without any signs of distress. In contrast, the 3MC-treated *Tiparp*^−/−^ mice died on or between days 8 to 16 (Figure 2A). The mice were either found dead in the morning on the day of death or had to be humanely euthanized due to poor health. An initial decrease in body weight of 3MC-treated *Tiparp*^−/−^ mice was followed by an increase after day 8. No significant differences in food intake were observed. This was most likely due to high variability among the animals and the lower number of surviving *Tiparp*^−/−^ mice as the 30-day experiment progressed. (Figure 2B,C). In each of the 3MC-treated *Tiparp*^−/−^ mice, abdominal distention was evident around days 5–6. This distention was due to the accumulation of fluid in the peritoneum, which may have accounted for the gradual increase in weight over time. All fluid samples obtained from these mice were milky white and viscous (Figure 2D).

**Figure 3:**
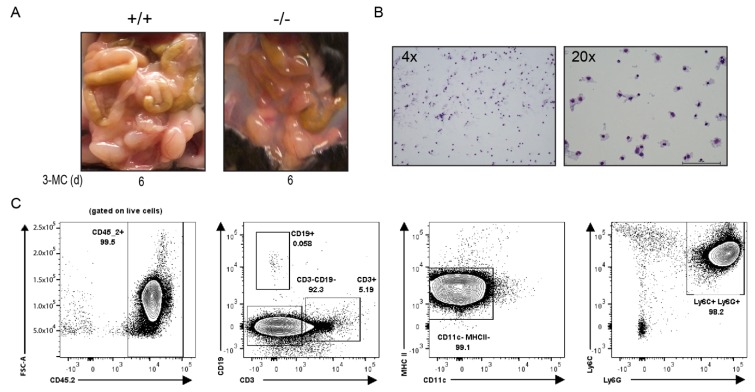
Wright Giemsa stain and flow cytometry analysis of the chylous ascites collected from the peritoneum of 3MC-treated mice on day 6. (**A**) Accumulation of fluid observed in 3MC-treated WT and *Tiparp*^−/−^ mice. (**B**) Cellular bodies present in the ascitic fluid. (**C**) To phenotype the cellular infiltrate, flow cytometry was used and identified the populations to be predominantly neutrophilic. Representative plot of four ascites samples showing a greater neutrophil population (Ly6C+, Ly6G+). Peritoneal fluid was stained directly. Almost all cells in fluid are hematopoietic (CD45.2+) and the majority are innate cell types (CD3- and CD19-). Of these innate cell types, they are not antigen-presenting cells (CD11c- and MHC II-).

We conducted a subsequent acute 6-day toxicity study to characterize the effects of 3MC on the *Tiparp*^−/−^ mice. Similar to that observed in the 30-day study, a translucent to milky white and viscous fluid accumulated in the peritoneal cavities of all 3MC-treated *Tiparp*^−/−^ mice on day 6 (Figure 3A). However, the samples were less viscous and more translucent compared with treated *Tiparp*^−/−^ mice in the 30-day study. Biochemical analyses of the collected fluid samples from the 3MC-treated *Tiparp*^−/−^ mice in the 30-day and 6-day study revealed high triglyceride levels and high protein concentrations. The triglyceride levels of the viscous fluid ranged from 322 to 1923 mg/dL, while protein levels ranged from 1.9 to 2.6 g/dL (Table 1). Wright Giemsa staining of the fluid revealed a high concentration of immune cells (Figure 3B). Flow cytometry was then used to phenotype the cells. The majority of the cells were hematopoietic (CD45.2+) and they were predominantly innate cell types since they were negative for CD3 and CD19, which are markers for T and B cells, respectively. These innate cells were not antigen-presenting cells (CD11c-, MHC II-) but were predominantly neutrophils (Ly6C+, Ly6G+) (Figure 3C). Based on these biochemical parameters, the fluid was similar to chylous ascites in humans [25], except for the predominance of neutrophils rather than the characteristic lymphocytic population observed in patients (Table 1).

**Figure 4:**
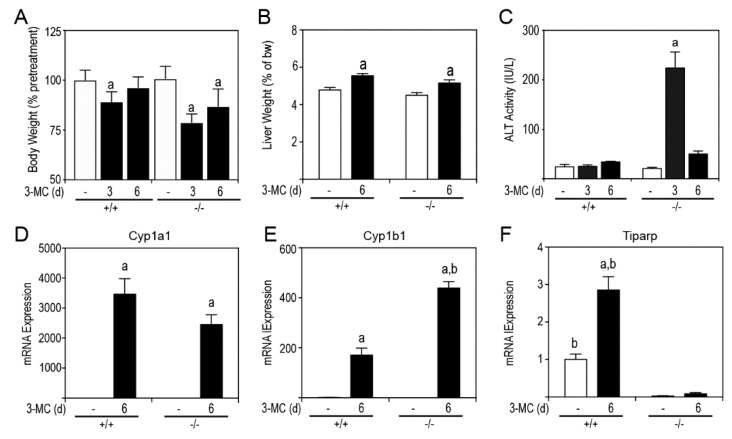
Six-day exposure to 100 mg/kg 3MC causes a transient increase in alanine aminotransferase (ALT) activity and increased hepatic Cyp1b1 but not Cyp1a1 expression levels. (**A**) Body weight. (**B**) Liver weight. (**C**) ALT activity levels. Gene expression levels of Cyp1a1 (**D**), Cyp1b1 (**E**), and Tiparp (**F**). Data represent the mean ± SEM; *n* = 3 for all genes. ^a^ *p* < 0.05 two-way ANOVA comparison between genotype-matched corn oil- and 3MC-treated mice and ^b^ *p* < 0.05 two-way ANOVA comparison between treatment-matched WT and *Tiparp*^−/−^ mice followed by a Tukey’s post hoc test.

Since liver toxicity is a contributing factor to chylous ascites, we examined the livers of 3MC- and corn oil treatments in WT and *Tiparp*^−/−^ mice. Significant reductions in body weight for both treated WT and *Tiparp*^−/−^ mice were observed at day 3, but only for *Tiparp*^−/−^ mice at day 6 (Figure 4A). Both 3MC-treated WT and *Tiparp*^−/−^ mice had increased liver weights (Figure 4B). 3MC-treated *Tiparp*^−/−^ mice had a significant, but transient, increase in serum alanine aminotransferase (ALT) activity (Figure 4C) on day 3, which returned to baseline on day 6. We next determined the hepatic AHR-regulated gene expression after 6 h and at day 6. The mRNA levels of Cyp1a1 were not significantly different between genotypes; however, Cyp1b1 mRNA levels were significantly greater in 3MC-treated *Tiparp*^−/−^ mice compared with similarly treated WT mice (Figure 4D,E).

**Figure 5:**
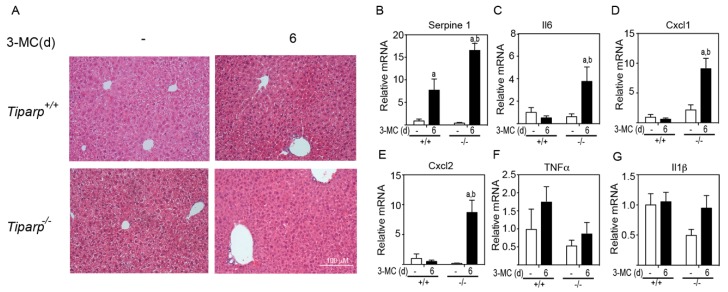
3MC induces increased inflammation and inflammatory cytokine levels in *Tiparp*^−/−^ compared with WT mice. (**A**) Hematoxylin & Eosin (H&E) staining of the liver lobe at day 6 after corn oil or 3MC treatment. 100× magnification. Scale bar represents 100 µm. Gene expression levels of Serpine 1 (PAI-1) (**B**), Il6 (**C**), Cxcl1 (**D**), Cxcl2 (**E**), Tnfα (**F**), and IL1β (**G**). Data represent the mean ± SEM; *n* = 3 for all genes. ^a^ *p* < 0.05 two-way ANOVA comparison between genotype-matched corn oil- and 3MC-treated mice and ^b^ *p* < 0.05 two-way ANOVA comparison between treatment-matched WT and *Tiparp*^−/−^ mice followed by a Tukey’s post hoc test.

The histopathology of the liver was examined to determine if there was any indication of liver toxicity or injury (Figure 5A). Hematoxylin & Eosin (H&E) staining of liver sections from corn oil-treated animals displayed an intact architecture with no migratory immune cells from the portal triad or the central vein. WT animals treated with 3MC showed mild microvesicular steatosis as evidenced by vacuolated hepatocytes. *Tiparp*^−/−^ animals treated with 3MC displayed an increase in the number of resident Küpffer cells in the sinusoids, signifying a mild inflammatory cell infiltration. In support of this increased inflammation, higher levels of the AHR-responsive inflammatory cytokines and chemokines Serpine 1, Il6, Cxcl1, and Cxcl2 were detected in 3MC-treated *Tiparp*^−/−^ mice compared with treatment-matched WT mice (Figure 5B–E). No significant differences in Tnfα and Il-1β levels were observed (Figure 5B–E).

**Figure 6:**
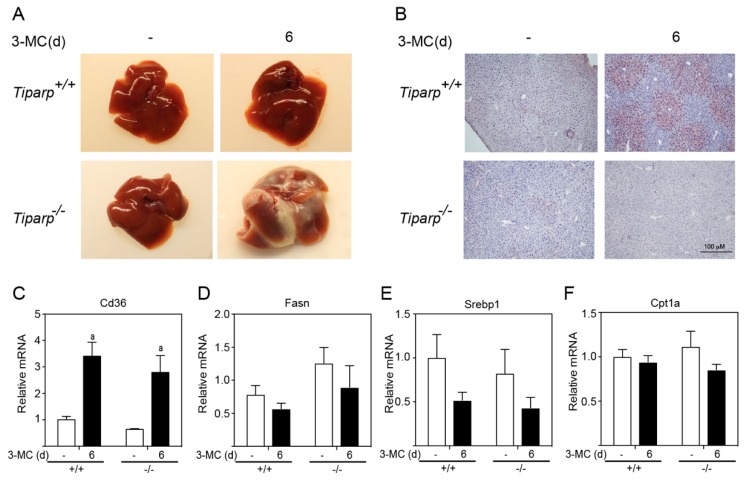
3MC induces hepatosteatosis in WT but not in *Tiparp*^−/−^ mice. (**A**) Gross liver images from corn oil- and 3MC-treated WT and *Tiparp*^−/−^ mice at day 6. (**B**) Oil Red O staining was conducted to visualize neutral fats. Gene expression levels of Cd36, (**C**), Fasn (**D**), Srebp1 (**E**), and Cpt1a (**F**). Data represent the mean ± SEM of inflammatory genes; *n* = 3. ^a^ *p* < 0.05 two-way ANOVA comparison between genotype-matched corn oil- and 3MC-treated mice followed by a Tukey’s post hoc test for multiple comparisons.

Gross liver images from WT corn oil- and 3MC-treated mice, as well as the *Tiparp*^−/−^ corn oil-treated animals, displayed a normal liver appearance with a rich red-brownish coloration (Figure 6A). All *Tiparp*^−/−^ mice treated with 3MC were found with white, lobular lesions encapsulating their livers. The source of this white infiltrate appeared to originate from the right lobe, where a white mass was also found in the area. To investigate the involvement of the AHR in hepatic steatosis, Oil Red O staining was conducted to visualize neutral fats. Both WT and *Tiparp*^−/−^ corn oil-treated mice exhibited normal liver histology (Figure 6B). Conversely, 3MC-treated WT mice had microvesicular fat accumulation around the central vein. However, this was not observed in the 3MC-treated *Tiparp*^−/−^ mice. No genotype differences in 3MC-induced lipid uptake transporter, Cd36, levels were observed (Figure 6C). No significant increases in the expression levels of genes involved in lipogenesis (Fasn, Srebp1) and β-oxidation (Cpt1a) were determined (Figure 6D–F).

**Figure 7:**
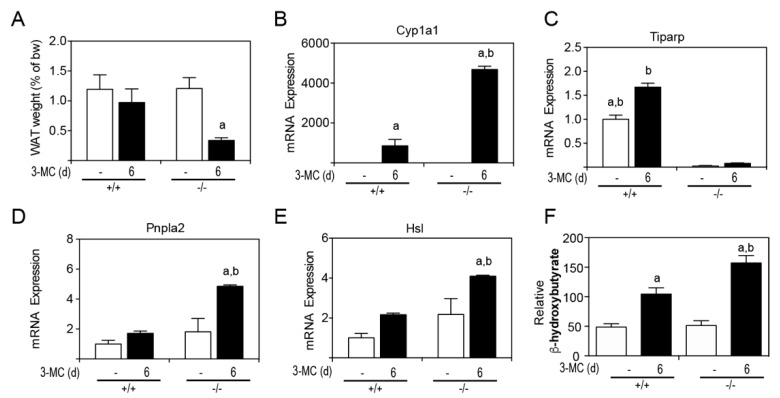
Increased AHR signaling and loss of white adipose tissue (WAT) weight levels in 3MC-treated *Tiparp*^−/−^ mice. (**A**) WAT weight was expressed as a percentage of total body weight on day 6. The mRNA expression levels of the AHR target genes Cyp1a1 (**B**), *Tiparp* (**C**), Pnpla2 (**D**), Hsl (**E**) and serum β-hydroxybutyrate (F) were measured. Data represent the mean ± SEM; with an *n* = 3 (**A**). ^a^ *p* < 0.05 two-way ANOVA comparison between genotype-matched corn oil- and 3MC-treated mice followed by a Tukey’s post hoc test for multiple comparisons and ^b^ *p* < 0.05 two-way ANOVA comparison between treatment-matched WT and *Tiparp*^−/−^ mice followed by a Tukey’s post hoc test.

Due to the lipid accumulation on internal tissues, perigonadal white adipose tissue (WAT) was removed and weighed at endpoint (Figure 7A). 3MC-treated *Tiparp*^−/−^ mice had an approximate 60% reduction in perigonadal WAT levels. The mRNA levels of the AHR target gene, Cyp1a1, were induced to a higher level in *Tiparp*^−/−^ mice compared with WT mice (Figure 7B). Tiparp mRNA levels were induced by 3MC treatment in WT mice but not in *Tiparp*^−/−^ mice (Figure 7C). Two lipases—Pnpla2 and Hsl—involved in triglyceride hydrolysis were also studied to examine their expression in WAT and involvement in lipid partitioning. *Tiparp*^−/−^ mice displayed increased mRNA expression levels of both Pnpla2 and Hsl in WAT compared to corn oil-treated controls or 3MC-treated WT mice (Figure 7D,E). Serum β-hydroxybutyrate levels were increased in 3MC-treated WT and *Tiparp*^−/−^ mice compared with control treated mice (Figure 7F). They were, however, significantly higher in *Tiparp*^−/−^ mice compared with WT mice, suggesting that the increased lipolysis and resulting free fatty acids are converted into energy and ketone body formation.

**Figure 8:**
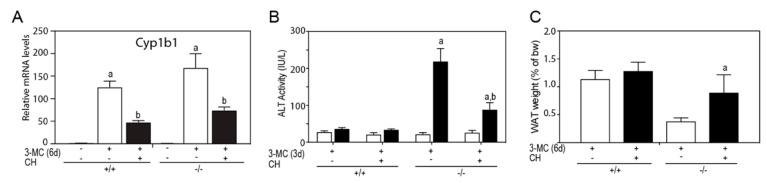
Cotreatment with CH223191 reduced 3MC-induced alanine aminotransferase (ALT) activity and epididymal adipose tissue loss. WT or *Tiparp*^−/−^ mice were cotreated with 100 mg/kg 3MC on day 1 and with 10 mg/kg CH223191 (CH) on days 1 and 3. (**A**) Hepatic Cyp1b1 mRNA expression levels. (**B**) ALT activity levels on day 3. (**C**) WAT weight was expressed as a percentage of total body weight on day 6. Data represent the mean ± SEM. ^a^ *p* < 0.05 two-way ANOVA comparison between (**A**) genotype-matched corn oil- and 3MC-treated mice, (**B**) genotype-matched day 0 and 3MC-treated day 3 mice, and (**C**) treatment-matched WT and *Tiparp*^−/−^ mice on day 6. ^b^ *p* < 0.05 two-way ANOVA comparison between WT and *Tiparp*^−/−^ mice treated with 3MC and 3MC + CH followed by a Tukey’s post hoc test.

To determine if AHR was mediating the 3MC-toxicity, *Tiparp*^−/−^ mice were cotreated with 3MC and the AHR antagonist, CH223191. Cotreatment with CH223191 reduced the 3MC dependent increase in Cyp1b1 mRNA levels (Figure 8A), reduced serum ALT activity (Figure 8B) and reduced epididymal WAT loss (Figure 8C). However, CH223191 cotreatment did not prevent 3MC-induced chylous ascites in *Tiparp*^−/−^ mice, but reduced the severity as indicated by significantly reduced triglyceride levels and increased clarity of the fluid (Table 2).

## 3. Discussion

Here we show that exposure to a high dose of 3MC is lethal to male *Tiparp*^−/−^ mice, but not to similarly treated WT animals. In previous studies, we reported that whole-body or hepatocyte-specific deletion of *Tiparp* results in an increased sensitivity to TCDD-induced hepatotoxicity, steatohepatitis and lethal wasting syndrome [13,24]. However, unlike TCDD, 3MC-treated *Tiparp*^−/−^ mice developed a chylous ascites-like condition, with evidence of hepatic inflammation, but without steatosis [25]. Treatment with the AHR inhibitor, CH223191, partially rescued the severity of the chylous ascites, implicating AHR as the mediator of the 3MC-induced toxic outcomes. Our data provide further evidence for the important role of TIPARP in the negative regulation of toxicant-induced AHR activity.

In agreement with previous work, we observed that a single IP injection of 100 mg/kg 3MC resulted in hepatic steatosis in C57BL/6 mice [11]. Hepatic steatosis was, however, not observed in similarly treated *Tiparp*^−/−^ mice. This may have been due to the higher induction of CYP1A1 expression that occurs in the absence of *Tiparp*, resulting in rapid hepatic 3MC metabolism; thus, preventing hepatic steatosis. In support of this, *Ahrr*^−/−^ mice show a delayed response to skin carcinogenesis caused by benzo[*a*]pyrene (B[*a*]P) due to increased Cyp1a1 levels in skin and more rapid metabolism and clearance of B[*a*]P [26]. However, no increases in hepatic Cyp1a1 levels were reported in liver, lung, and heart, suggesting that the loss of Ahrr expression favors the detoxification of carcinogens via increased Cyp1a1 levels in some but not all tissues. Whether *Tiparp*^−/−^ mice also show a shift in the metabolism that favors the detoxification of chemical carcinogens has not been determined.

Chylous ascites is defined as a build-up of lymph within the abdomen due to obstruction in the abdominal lymphatic system [27]. Normally, the lymphatic system returns interstitial fluid and proteins to the venous circulation via lymphatic vessels to the lymph nodes. These channels drain into the cisterna chyli at the start of the thoracic duct; however, damage or obstruction to this network can lead to a chyle leak. During such a situation, the lymph fluid becomes milky and viscous due to the conversion of long-chain triglycerides into free fatty acids and monoglycerides, there is an increase in protein content, and an influx of myeloid and lymphoid cell populations [25]. In humans, chylous ascites predominantly contain lymphocytes with a small number of neutrophils [28]. If a predominance of neutrophils is observed, it would be suggestive of peritonitis that may result from an infection [29]. Moreover, AHR activation is known to increase the number of neutrophils recruited to infected tissues, such as lung airways during influenza infection [30]. The chylous ascites observed in the 3MC-treated *Tiparp*^−/−^ mice was very similar in composition to that observed in humans except for the predominance of neutrophils. Gram staining of the peritoneal fluid was negative for the presence of bacteria (data not shown). Thus, the reason for the neutrophilia in the peritoneal cavity is unknown, but may be in part due to increases in the neutrophil chemoattractants Cxcl1 and Cxcl2, which were elevated in livers of 3MC-treated *Tiparp*^−/−^ mice. However, their serum or peritoneal fluid levels were not determined in our study. Since chylous ascites was not observed in TCDD-treated *Tiparp*^−/−^ mice, the recruitment of neutrophils to the peritoneal cavity is influenced by the nature of the AHR ligand and not a simple result of AHR activation in *Tiparp*^−/−^ mice.

Treatment of *Tiparp*^−/−^ mice with either 10 µg/kg TCDD [13,24] or 100 mg/kg 3MC (the present study) is lethal. However, the effect of each AHR ligand differs with respect to the observed toxicity and the cause of death. TCDD-treated animals display increased sensitivity to hepatotoxicity and wasting syndrome, whereas 3MC-treated animals present with chylous ascites and only mild liver toxicity. Reduced levels of epididymal WAT and increased expression of lipolytic enzymes were consistently observed after treatment with either AHR ligand [13,24]. 3MC-treated *Tiparp*^−/−^ mice lost significant body weight and epididymal WAT without any reduction in food intake, suggesting that *Tiparp* loss may affect the efficiency of intestinal fat and or nutrient absorption perhaps due to an obstruction in the lymph. The lack of efficient lipid absorption could explain the increase in lipolysis, which would be needed to provide energy that was not being obtained from the food. This is supported by increased serum β-hydroxybutyrate levels, suggesting increased energy from β-oxidation in the liver.

Although this is the first report that 3MC exposure causes chylous ascites, other studies using a variety of transgenic animal models have observed a similar phenotype. In a transgenic mouse model where overexpression of vascular endothelial growth factor (VEGF)-C was induced in adipocytes, chylothorax was observed within seven days of doxycycline treatment in drinking water which led to in overexpression of VEGF-C [31]. Lymphatic vessels in VEGF-C transgenic mice were enlarged and allowed for retrograde flow of milky, triglyceride-rich chyle from the thoracic duct back into the originating lymphatics and, consequently, into the thoracic cavity due to weakened valves and other lymphatic abnormalities promoted by VEGF-C overexpression. The deletion of RASA1, a Ras GTPase-activating protein that negatively regulates lymphatic vessel growth, resulted in a lymphatic vessel disorder characterized by extensive lymphatic vessel hyperplasia, dilation, leakage, and early lethality caused by chylothorax [32]. Patients with a mutation in RASA1 are at a higher risk of developing Parkes–Weber syndrome, which presents itself as a disease with upper and lower extremity lymphedema with some cases of chylothorax and/or chylous ascites [33]. Exposure to TCDD or 3MC has been demonstrated to upregulate VEGF expression [34]. Moreover, adult *Tiparp*^−/−^ mice show evidence of vascular defects [35]. Together with the increased sensitivity of *Tiparp*^−/−^ mice to AHR ligands, the activation or downregulation of components in other signaling pathways may lead to the malformation of the lymphatic system, resulting in the accumulation of extravasated fluid. However, whether the accumulation of chylous fluid in the *Tiparp*^−/−^ mice is due to the obstruction of the lymphatics or a defect in dietary and endogenous lipid absorption and/or metabolism remains unknown.

In summary, we show that 3MC-treated *Tiparp*^−/−^ mice display an increased sensitivity to 3MC-induced toxicity and lethality, further supporting the role of Tiparp as an important negative regulator of AHR-mediated responses. However, we cannot exclude the possibility that various 3MC metabolites are also involved, since the increased Cyp1a1 levels in 3MC-treated *Tiparp*^−/−^ mice would result in elevated levels of 3MC-derived metabolites. Future studies evaluating alternative routes of administration, lower doses of 3MC, other PAHs and/or AHR ligands, and effects in additional genetically modified mouse models will be needed to determine the etiology of the 3MC-induced chylous ascites in the absence of Tiparp expression.

## 4. Materials and Methods 

### 4.1. Chemicals and Biological Reagents

For chemical treatments, a 100-mg vial of 3-methylcholanthrene (3MC) was purchased from Sigma-Aldrich (St. Louis, MO, USA) at an HPLC purity of >97.5%. Dimethyl sulfoxide (DMSO) and CH223191 were also purchased from Sigma-Aldrich. One-hundred percent pure corn oil (CO) was purchased from a local grocer. A 10 mg/mL stock of 3MC was made and this solution was made fresh before injection and disposed of after 30 days. Liver sections for H&E staining were preserved in neutral buffered 10% formalin solution (Sigma-Aldrich), and liver sections for Oil Red O were suspended in VWR^®^ Clear Frozen Section Compound (Radnor, PA, USA) to embed tissues for cryosectioning. The Infinity™ ALT Liquid Stable Reagent was purchased from Fisher Diagnostics (Middletown, VA, USA) for use in the in vitro determination of ALT activity in mouse serum.

### 4.2. Animals

*Tiparp*Gt(ROSA)79Sor mutant mice (stock number: 007206) were purchased from Jackson Laboratories (Bar Harbor, ME, USA) and have been previously described [13,35]. The animal colony was maintained by breeding heterozygotes. Only WT (*Tiparp*^+/+^) and *Tiparp*^−/−^ mice were used in experiments. Animals were housed in the Division of Comparative Medicine at the University of Toronto (Toronto, ON, Canada). The temperature was constant at 21 °C; a maintained light–dark cycle (12 h and 12 h); humidity within the facility was controlled; and standard rodent chow and sterile water were provided ad libitum. All procedures and experiments conducted were in accordance with the principles set by the Canadian Council on Animal Care guidelines and approved by the Local Animal Care Committee (protocol # 20010338) on the 9th of September 2014 at the University of Toronto.

### 4.3. MC and CH223191 Treatment

Seven-to-nine week old male *Tiparp*^+/+^ and *Tiparp*^−/−^ mice were given a single intraperitoneal injection of 100 mg/kg body weight of 3MC dissolved in corn oil. Control (corn oil) mice received an equivalent volume of corn oil corrected for body weight. For 3MC and the AHR antagonist CH223191 cotreatment studies, mice were injected with 100 mg/kg 3MC and 10 mg/kg CH223191 dissolved in DMSO or an equivalent volume of DMSO as control. The animals received a second IP injection of CH or DMSO on day 3. Solutions were heated to 37 °C and vortexed to ensure solubilization of the compound prior to treatment. Mice were monitored daily and proper personal protective equipment was implemented for the handling of 3MC-treated animals. If considered endpoints were met at any moment during the experiment, humane intervention was implemented to prevent or relieve unnecessary pain and distress. Suggested endpoints include body weight loss exceeding 20% of normal body weight as measured on day 0, severe lethargy and reluctance to move when provoked, hunched or abnormal posture, severe dehydration or malnutrition, and signs of severe discomfort. If these ailments could not be alleviated through preventative measures, then the mice were humanely euthanized. Animals were supplied with standard chow (Teklad Global Diet^®^ 2018; 18% protein, 6% fat) in the form of pelleted food from Harlan Laboratories (Indianapolis, IN, USA).

### 4.4. Body and Food Weight Measurements

Mice and food pellets were weighed daily in the morning and values were recorded throughout the study. Body weight was taken after stabilization of weight fluctuation by the scale. For food intake measurements, ~100 g of intact food pellets were placed on the top wire feeder and weighed on Day-1 for the calculation of the baseline value (day 0). All pellets on top of the wire feeder as well as any residual pieces on the cage floor were accounted for in the daily food intake measurement. Measurements recorded on each subsequent day were subtracted from the previous day’s recorded value to provide the daily food intake value. Body weight and food intake values were normalized to baseline and graphed as an increase or decrease from day 0.

### 4.5. Blood Collection and ALT analysis

Blood was collected from the saphenous vein of the hind leg. Approximately 100 µL of blood was collected in a Microvette^®^ 200 Z-Gel tube, and this was conducted on day 0 (before treatment for baseline values), day 3, and day 6. Blood samples were placed at room temperature for a minimum of 30 min for coagulation. The sample can then be centrifuged at 10,000 rpm for 5 min to separate the serum, which was subsequently collected and stored at −80 °C until ALT analysis. The Infinity™ ALT (GPT) Liquid Stable Reagent (Fisher Diagnostics) was warmed to 37 °C for optimal assay conditions. Samples were processed in duplicates. Immediately before assay measurements, 160 μL of the ALT reagent was aliquoted into each well. The constant temperature was set at 37 °C within the BioTek Synergy™ MX multi-mode microplate reader (BioTek Instruments) and kinetic measurements were taken at an absorbance of 340 nm each minute for a total duration of 15 min. Activity levels were adjusted with the recommended factor. Values were then plotted against time and the average of the two slopes was obtained.

### 4.6. β-Hydroxybutyrate Levels

β-hydroxybutyrate levels were measured using an assay kit (Sigma-Aldrich). A serum sample volume of 10 μL was used and was directly added onto a 96-well plate. The assay was performed according to the supplier’s specifications and preparation instructions. Absorbance was measured at 450 nm.

### 4.7. RNA Extraction and Isolation

Mice were humanely euthanized by cervical dislocation and the whole liver was washed in ice-cold PBS, dried quickly on absorbent paper, and recorded for tissue weight. Epididymal WAT was removed from the perigonadal region washed in ice-cold PBS and weighed. All tissues were flash-frozen immediately in liquid nitrogen after recording tissue weights. Collected fluid samples were prepared on microscope slides before storing at −80 °C. For RNA isolation from liver, approximately 50 mg of frozen liver was homogenized in 500μL of TRIzol^®^ reagent. Samples were incubated at room temperature to allow for the complete dissociation between complexes before the addition of chloroform. The samples were vigorously vortexed and centrifuged at 13,000 rpm for 15 min at 4 °C for phase separation. The RNA, located in the upper aqueous phase, was added with 70% ethanol of equal volume. The lysate was thoroughly mixed and transferred into RNA binding columns supplied by the Aurum™ Total RNA Mini Kit. Once the RNA was eluted, these tubes were placed directly on ice. RNA concentration, purity, and quality were measured using the spectrophotometer at a 40-fold dilution in water. Extracted liver RNA samples were adjusted to 50 ng/μL with the addition of DNase/RNase-free distilled water.

### 4.8. cDNA Synthesis and Gene Expression Analyses

For the synthesis of cDNA from RNA, 10 μL of 500 ng normalized liver was reverse transcribed using SuperScript^®^ III and its components. The reaction mix consisted of 4 μL 5× First Strand buffer, 0.1 mM DTT, 50 mM random hexamers, 10μM dNTP mixture, distilled water, and SuperScript^®^ III in a total reaction volume of 20 μL per sample. Using the MJ Cycler Software 2.0 (Bio-Rad) on the Bio-Rad Chromo4™ DyadDisciple™, the cDNA synthesis reaction involved an initial 1 h incubation at 50 °C followed by a 15 min at 70 °C to inactivate the enzyme. The synthesized reaction was then diluted with DNase/RNase-free distilled water. The qPCR reaction was prepared using SsoFast EvaGreen^®^ SYBR Supermix, and 10 μM forward and reverse primer verified with NCBI Primer-BLAST (Bethesda, MD, USA). Technical duplicates were performed for each target gene transcript and normalized to the tata binding protein (Tbp) mRNA content. Reactions were performed on the Bio-Rad Chromo4™ DyadDisciple™ with the following conditions; 95 °C for 3 min and 45 cycles of 95 °C for 5 s for denaturation and 60 °C for 20 s. Data were analyzed using the Opticon Monitor™ 3 software (Bio-Rad) and fold changes were computed by the comparative cycle threshold (ΔΔCT) method and normalized to corn oil-treated WT controls.

### 4.9. Tissue Histology

To prepare slides for Hematoxylin & Eosin (H&E) staining, liver sections obtained from the animal dissections were freshly fixed in neutral buffered 10% formalin solution before processing and paraffin embedding. In this procedure, the liver was sectioned into 5-µm-thick segments. To prepare slides for Oil Red O staining, liver samples were suspended in VWR^®^ Clear Frozen Section Compound and flash-frozen in liquid nitrogen as previously described [13]. Sections of 5-µm-thick tissue-embedded ribbons were sliced using a cryostat and adhered onto a glass slide. The aforementioned procedures were services provided at Princess Margaret Hospital (Toronto, ON, Canada) of the University Health Network. For each slide, representative images of the cell population were obtained at 40×, 100×, and 200× magnification.

### 4.10. Wright Giemsa Stain

Peritoneal ascites was suspected from the observable distension of the abdomen, the peritoneum was carefully slit for the insertion of a 1cc Luer-slip syringe and withdrawal of the fluid for inspection and analysis. An aliquot of the sample was smeared as a thin film across a microscope slide using aseptic techniques over an open flame. The sample was allowed to air-dry before fixing in 100% methanol. The fixed sample was flooded with modified Accustain^®^ Wright Giemsa stain and an equal volume of distilled water was added to the stain. For visualization, Wright Giemsa-stained slides were imaged using a brightfield microscope and Nikon NIS-Elements Viewer imaging software. For each slide, representative images of the cell population were obtained at 40×, 100×, and 200× magnification.

### 4.11. Flow Cytometry

Peritoneal ascites were isolated and stained directly using fixable viability dye in eF506 (eBioscience, San Diego, USA) and for surface markers using the following antibodies; anti-mouse CD45.2 in FITC (clone: 104; eBioscience), anti-mouse CD3ε in PE-Cy7 (clone: 145-2C11; eBioscience), anti-mouse CD19 in BV605 (clone: 6D5; BioLegend, San Diego, USA), anti-mouse MHC class II in e450 (clone: AF6-120.1; eBioscience), anti-mouse CD11c in AF700 (clone: N418; eBioscience), anti-mouse Ly6C in PerCP-Cy5.5 (clone: HK1.4; eBioscience), and anti-mouse Ly6G in APC (RB6-8C5; eBioscience). Samples were analyzed using LSRFortessa (BD Biosciences, San Jose, USA) and FlowJo (TreeStar Inc., Ashland, USA) software.

### 4.12. Statistical Analysis

Daily body weight and food intake measures are expressed as the mean ± standard error of the mean (SEM) across all animals and analyzed by repeated measures two-way analysis of variance (ANOVA) with a Tukey’s post hoc statistical test for multiple comparisons between day-matched mice. A log-rank (Mantel–Cox) test was used in the survival curve analyses to determine significance (*p* < 0.05) between groups. In all other results, a two-way analysis of variance (ANOVA) followed by Tukey’s multiple comparisons test was used to determine statistical significance (*p* < 0.05). All data were graphed and analyzed using GraphPad Prism 6 statistical software (San Diego, CA, USA) using grouped measures.

## Figures and Tables

**Table 1:** Characteristics of chylous ascites observed from 3MC-treated *Tiparp*^−/−^ mice. Values for the clinical diagnosis of chylous ascites were adapted from Cárdenas and Chopra (2002) [25]. Values in parentheses indicate the number of observations over the number of total observations.

Measure	Clinical Diagnosis of Chylous Ascites	*Tiparp*^−/−^ Mice Days 8–16	*Tiparp*^−/−^ Mice Day 6
**Appearance**	Milky white and cloudy	Milky white and cloudy (7/7)	Milky white and cloudy (4/6) White and translucent (2/6)
**Triglyceride Level**	>200 mg/dL	48–2065 mg/dL	322–1923 mg/dL
**Cell Population**	Lymphocytes	Neutrophils	Neutrophils
**Total Protein**	1.1–7.0 g/dL	1.9–5.1 g/dL (mean: 3.8 g/dL)	1.9–2.6 g/dL (mean: 2.2 g/dL)

**Table 2:** Characteristics of ascites observed after 3MC and CH223191 cotreatment compared to 3MC alone in *Tiparp*^−/−^ mice. Values in parentheses indicate the number of observations over the number of total observations.

Measure	*Tiparp*^−/−^ Mice Day 6 3MC	*Tiparp*^−/−^ Mice Day 6 3MC + CH223191
**Appearance**	Milky white and cloudy (4/4)	Clear fluid (1/4) white and milky (3/4)
**Triglyceride Level**	560–1670 mg/dL (mean: 924 mg/dL)	135–1264 mg/dL (mean: 853 mg/dL)
**Cell Population**	Neutrophils	Neutrophils
**Total Protein**	3.4–4.0 g/dL (mean: 3.6 g/dL)	3.5–4.0 g/dL (mean: 3.7 g/dL)

## Abbreviations

3MC	3-methylcholanthrene
AHR	aryl hydrocarbon receptor
AHRE	AHR response element
ARNT	AHR nuclear translocator
ARTD	ADP-ribosyltransferase diphtheria toxin-like
B[a]P	benzo[a]pyrene
CYP1A1	cytochrome P450 1A1
IP	intraperitoneal
PAH	polycyclic aromatic hydrocarbon
PARP	poly-ADP-ribose polymerase
TCDD	2,3,7,8-tetrachlorodibenzo-*p*-dioxin
TIPARP	TCDD-inducible poly-ADP-ribose polymerase
WAT	white adipose tissue
